# Emergence of new-onset psychotic disorder following recovery from LGI1 antibody-associated limbic encephalitis

**DOI:** 10.1136/bcr-2016-218328

**Published:** 2017-03-31

**Authors:** Thomas A Pollak, Nick Moran

**Affiliations:** 1 Department of Psychosis Studies, Institute of Psychiatry, Psychology and Neuroscience, King's College London, London, UK; 2 King's College Hospital NHS Foundation Trust, London, UK

## Abstract

Neuronal autoantibodies targeting cell surface antigens have been described in association with autoimmune encephalitides which frequently feature psychosis and other psychiatric disturbances alongside neurological signs and symptoms. Little has been written however about the long-term psychiatric status of individuals following recovery from the acute phase of autoimmune encephalitis, despite case series and anecdotal evidence suggesting this may be a cause of considerable disability. Here, we describe a man aged 58 years with no psychiatric history who developed a severe and acute psychotic disorder following resolution of a protracted course of limbic encephalitis associated with antibodies to leucine-rich glioma inactivated 1 protein. No indications of a gross ongoing inflammatory or encephalopathic process were present at presentation of his psychosis. Possible aetiologies of his acute psychosis are discussed. This case highlights the importance of ongoing psychiatric follow-up of patients following an episode of autoimmune encephalitis.

## Background

Autoimmune encephalopathy syndromes associated with autoantibodies to the voltage-gated potassium channel (VGKC) or its complexed proteins were first described in 2001.^1^ Subsequently, the spectrum of neurological disorders associated with VGKC complex antibodies has expanded considerably to encompass multiple central and peripheral nervous system manifestations.^2^ However, in some cases, VGKC complex antibodies, particularly when present at low titre, may not be pathogenic.^3^ In 2010, the receptor-associated proteins like contactin-associated protein-like 2 (CASPR2) and leucine-rich glioma inactivated 1 (LGI1) were identified as the antigenic targets in most cases of VGKC antibody-associated syndromes.^4^ The most characteristic syndrome is limbic encephalitis, frequently featuring confusion, memory impairment, psychiatric symptoms and seizures.^5^ LGI1 antibody-associated encephalitis is frequently preceded by a highly characteristic seizure semiology termed ‘faciobrachial dystonic seizures’.^5^

VGKC antibodies that do not have LGI1 or CASPR2 as their target antigen have recently been identified as having largely intracellular targets, making their pathogenicity unlikely.^6^

Case reports of individuals with VGKC complex autoantibodies with predominantly psychiatric presentations have been published and VGKC complex autoantibodies have recently been identified in a proportion of all individuals presenting to psychiatric services with psychosis.^7–9^

The outcome of VGKC complex-associated limbic encephalitis is variable and shows some antigen specificity: LGI1 encephalitis is associated with greater cognitive deficits and rates of hippocampal atrophy than CASPR2 encephalitis.^10^ Although the natural history of the disease is likely to be self-limiting if left untreated with immunotherapy,^11^ and the extent of cognitive deficits can be limited by early initiation of immunotherapy;^12^ ^13^ only around one-third of patients recover their baseline cognitive function.^14^ Relapse of LGI1 encephalitis occurs in 35% of patients, sometimes as long as 8 years after initial presentation.^15^

The published data on long-term psychiatric outcome following VGKC complex-associated limbic encephalitis generally, or LGI1 encephalitis more specifically, are scant but suggests that there may be significant residual psychiatric symptoms and that de novo psychiatric features, including anxiety and affective and impulse-control disorders, may develop.^16^

## Case presentation

In August 2013, a right-handed man aged 57 years was admitted to hospital with a week's history of confusion, personality change and a witnessed generalised tonic–clonic seizure. For 4 months before the admission, he had been noted by his wife to be chanting in his sleep; the chants made reference to death and dying. Nocturnal arm twitching was noted for 2 months before admission and leg muscle twitching had been noted for about a year. His medical history was notable for degenerative lumbar canal stenosis treated by surgical decompression in 2010, which was complicated by a dural tear and infection. He had a family history of Cowden syndrome.

In the ward, he had three further generalised seizures. An MRI brain revealed left medial temporal lobe inflammatory changes. Cerebrospinal fluid (CSF) analysis showed lymphocytosis (lymphocyte count 10, polymorphonuclear neutrophil count 0, red cell count 334, protein 1.03 g and glucose 3.9). CSF PCR for herpes simplex virus was sent but, unfortunately, lost. He was treated empirically with acyclovir, ceftriaxone and levetiracetam with improvement and was discharged home.

Two to 3 weeks after discharge, his condition began to deteriorate, with worsening memory, visual hallucinations, delusions of persecution by unknown assailants, continued nocturnal chanting and frequent déjà vu. He would put metal cutlery into the toaster and walk off into busy roads. He was noted to have brief paroxysmal movements involving his neck and arms. By 3 months after initial presentation, he required constant supervision. Test for VGKC complex antibodies on a serum sample taken on the initial admission returned positive with a titre of 1834 pmol/L. He was readmitted to hospital. Examination was notable for disorientation to place, echopraxia and bilateral weak ankle dorsiflexion (a sequel of lumbar canal stenosis). He was hyponatraemic.

## Investigations

**Figure 1:**
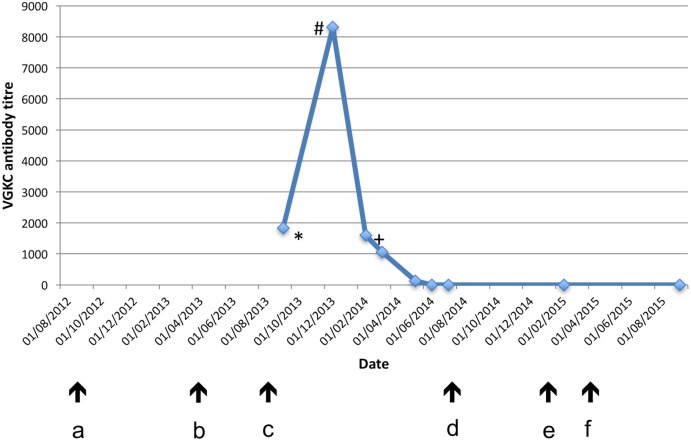
Voltage-gated potassium channel antibody titre: relation to neuropsychiatric symptoms and treatment. (a) Onset of leg muscle ‘twitching’; (b) onset of nocturnal behavioural disturbance; (c) onset of florid encephalopathy, including confusion, hallucinations and seizures; (d) discharge from hospital with resolving confusion, seizure freedom; (e) onset of severe, acute psychosis; (f) recovery from acute psychosis on antipsychotic medication. *LGI1 antibody positive; #intravenous immunoglobulins and plasma exchange started; +cyclophosphamide started.

The following were negative: ANA, ANCA, ENA, anti-GAD antibodies, HIV I and II antibodies, hepatitis B and C and syphilis serology tumour markers (AFP, CEA and CA199). The VGKC complex antibody titre had risen to 8310 pmol/L (figure 1). A scrotal ultrasound scan and a CT of the chest, abdomen and pelvis revealed no malignancy. A repeat MRI brain showed abnormal T2/FLAIR hyperintensity in the left caudate, anterior putamen and insula with mild swelling. Prolonged EEG showed generalised slowing with frequent subclinical seizures bilaterally and one secondary generalised seizure. The twitching episodes in sleep were not associated with abnormal EEG correlates. He was confirmed by staff to be experiencing clinical faciobrachial dystonic seizures as well as generalised tonic–clonic seizures. Subsequent analysis, in 2016, of his first VGKC complex antibody-positive serum sample was positive for LGI1 antibodies.

## Treatment

He received 3 days intravenous methylprednisolone followed by plasma exchange and 5 days intravenous immunoglobulins for presumed LGI1 antibody-mediated encephalitis. Levetiracetam was switched to zonisamide and sodium valproate. He made a significant recovery with cessation of observable seizures and was discharged home on a reducing course of prednisolone at an initial dose of 60 mg daily.

## Outcome and follow-up

Six months after presentation, he underwent neuropsychological testing, which highlighted significantly impaired verbal memory (1st centile) and below-expected performance on processing speed and executive function. His community rehabilitation team described a slow functional recovery and made note of moderate anxiety and depressive symptoms.

Seven months after initial presentation, he was readmitted for recurrent seizures and increasing confusion. His prednisolone dose at the time had been tapered to 30 mg daily. The VGKC complex antibody titre was 1050 pmol/L. He was treated with intravenous methylprednisolone 500 mg once daily for 3 days followed by plasma exchange and then pulsed intravenous cyclophosphamide (15 mg/kg weekly at time 0, 2 and 4 weeks). He was assessed by the neuropsychiatry team who noted there was no psychosis. He required intensive rehabilitation input and was eventually discharged in August 2014, a year after initial presentation.

Five months later (∼17 months after presentation), he was noted by his neuropsychiatry team to be experiencing visual hallucinations, aggressive outbursts, paranoid ideas and the belief that in the past he had been infected by many small creatures called ‘mots’ who had been sent by a shadowy man in black and were eating him from the inside of his body. It was reported by his family that these symptoms had been ongoing for 2 months. Over the following days, his paranoid behaviour and aggression to his family worsened and he was readmitted to hospital for investigation of a presumed relapse of encephalitis. On admission, he was fully alert and orientated and no new neurological signs or seizures were noted. He became increasingly agitated and expressed persecutory ideas about hospital staff, refusing to eat and drink in the belief that food and fluid had been poisoned.

EEG was within normal limits and MRI showed an atrophic left hippocampus with no new inflammatory changes. Serum VGKC complex antibodies were negative.

Owing to worsening psychosis and increasing risk, he was admitted to a psychiatric ward where he initially required intensive 1:1 nursing. He was noted to be paranoid, grandiose (expressing delusions of wealth and plans to ‘fix’ the National Health Service) and tangential with aggressive outbursts. He was started on risperidone and regular benzodiazepines and his psychotic symptoms improved over the following weeks. After a 2-month admission, he had made a full recovery and he was discharged on risperidone 4 mg.

He has ongoing cognitive difficulties and he and his carer describe prominent apathy, with occasional nightmares and health-related anxiety. He remains under the care of a community mental health team and has had no recurrence of any psychotic symptoms on a reducing dose of risperidone (currently 3 mg), fluoxetine 20 mg and low-dose benzodiazepines.

## Discussion

This is the first description of a de novo psychotic illness developing following autoimmune encephalitis in a patient with no previous psychiatric history. VGKC complex autoantibody-associated encephalitis commonly presents with psychotic and confusional symptoms as part of a wider constellation of psychiatric and neurological symptoms,^17^ as it did in the current case. Remarkably, however, VGKC complex antibodies were subsequently undetectable during a severe psychotic deterioration characterised by agitation, persecutory delusions, grandiosity and thought disorder but no concurrent confusion. MRI and EEG did not support an ongoing encephalitic process.

Possible aetiologies for the acute psychotic episode include: (1) pathogenic antibodies being present in CSF but not in serum (ie, atypical relapse of LGI1 encephalitis); (2) a manifestation of other postencephalitic neurological changes; or (3) a response to the stress of illness.

We consider an atypical relapse of LGI1 encephalitis to be unlikely, given the swift resolution of his psychosis with antipsychotic treatment and the lack of any neurological symptoms or paraclinical evidence of current encephalitis. Further, analysis of paired serum-CSF samples suggests intrathecal synthesis is rare in encephalitis associated with antibodies to the VGKC complex.^18^

Given mounting evidence for the use of positron emission tomography (PET) in the assessment of autoimmune encephalitis,^19–21^ it is possible that PET may have a future role in the assessment of cases such as this, where demonstration of continued hypermetabolic activity, even in the absence of positive MRI and EEG findings, could indicate ongoing inflammation.

Postencephalitic psychosis has been described before^22^ but not in association with autoimmune encephalitis. It is likely, however, that many cases of what in the past was called ‘idiopathic’ (or even ‘viral’) encephalitis may have autoimmune aetiologies.^23^ At an epidemiological level, autoimmune diseases associated with brain-reactive autoantibodies increase the risk of the subsequent development of non-affective psychoses and bipolar disorder.^24^ We therefore consider it likely that his psychosis was aetiologically related to his autoimmune encephalitis, perhaps in a manner analogous to postencephalitic epilepsy, although the mechanism remains unclear. Notably, hippocampal volume loss is a consistently identified vulnerability factor for non-organic psychosis.^25^ To this extent, the initial delay in diagnosis and treatment may have been a factor in his outcome and possibly the development of psychosis.

The patient had a strong family history of Cowden syndrome but had not been tested himself. The syndrome is caused by a dominant mutation in the PTEN tumour suppressor gene and typically manifests with malignancy and cutaneous manifestations. No malignancy was found in the patient as part of his initial workup following his initial presentation with LGI1 encephalitis, although he did have a number of cutaneous manifestations typical of Cowden syndrome. PTEN mutations are associated with an increased liability to autoimmune disorders, although have not to date been described in association with autoimmune encephalitis.

This case report demonstrates the importance of monitoring for de novo psychiatric symptoms following resolution of autoimmune encephalitis. Systematic collection of data relating to the mental health of this group is urgently required. In combination with the evidence for persistent psychiatric and cognitive sequelae in the postacute phase,^16^ we consider that psychiatric follow-up ought to be the norm rather than the exception in cases of autoimmune encephalitis.

**Learning points**

- Clinicians should have a high index of suspicion for an autoimmune aetiology in patients with an encephalopathic presentation, particularly those in whom an initial infective workup is negative.
- Leucine-rich glioma inactivated 1 (LGI1) antibody encephalitis can present with unusual behaviours including psychotic symptoms, usually together with neurological symptoms including seizures. Faciobrachial dystonic seizures are highly characteristic of the disease.
- Recovery from LGI1 antibody encephalitis can be protracted and may require multiple immunotherapies. Cognitive deficits following recovery are common and relapses occur in a third of patients, mandating ongoing neurological follow-up.
- Patients with this and other autoimmune encephalitides should be followed-up psychiatrically as new-onset psychiatric disorders, including psychosis, may occur and can be severe.
- Differentiating ongoing encephalitis from new-onset psychiatric disorder can be challenging but is essential to guide appropriate choice of treatment.
